# COVID-19 during pregnancy: case report and literature review

**DOI:** 10.11604/pamj.2023.45.9.26980

**Published:** 2023-05-04

**Authors:** Nadia Slimene, Cyrine Bennasrallah, Hela Abroug, Ines Charrada, Wafa Dhouib, Imen Zemni, Manel Ben Fredj, Chawki Loussaief, Asma Belguith Sriha

**Affiliations:** 1 Department of Family Medicine, University of Monastir, Monastir, Tunisia,; 2 Department of Epidemiology and Preventive Medicine, Fattouma Bourguiba University Hospital, University of Monastir, Monastir, Tunisia,; 3 Department of Endocrinology and Internal Medicine, Fattouma Bourguiba University Hospital, University of Monastir, Monastir, Tunisia,; 4 Department of Infectiology, Fattouma Bourguiba University Hospital, University of Monastir, Monastir, Tunisia

**Keywords:** COVID-19, outbreak, maternal, pregnancy, case report

## Abstract

The current coronavirus disease 2019 (COVID-19) pneumonia pandemic, caused by the newly discovered coronavirus is a serious public health emergency and a highly infectious disease. Evidence to date suggests that there are groups of people who are at a higher risk of getting severe COVID-19 disease such as pregnant women and their fetuses. We reported 4 cases of pregnant women with COVID-19 admitted in the national containment center, Tunisia (3 imported cases and one local case). The age range of the patients was 27-35 years and the range of gestational weeks at admission was 16 weeks to 32 weeks. None of the patients had underlying diseases. All four cases were totally asymptomatic and presented no complications. Two of them gave birth one by vaginal and the other by cesarean delivery, neonates presented no symptoms and no adverse outcomes. The current report does not present significant differences in the disease prognosis in the pregnant women´s group compared with the general women´s population. Careful observation, data collection and consecutive research are necessary.

## Introduction

The coronavirus disease 2019 (COVID-19) has led to a worldwide public health problem. The first cases were reported in Wuhan, China, in December 2019, followed by a large outbreak across the country [1]. On March 11^th^, 2020, The World Health Organization (WHO) declared the COVID-19 outbreak a pandemic [2].

Coronavirus is challenging the healthcare systems worldwide [2]. The risk of COVID-19 to mothers and newborns is widely uncharted [3]. The pregnant state with hormone level modifications and a slightly compromised immune system may predispose to a higher risk for both mothers and fetuses [4]. Some reports indicate that outcomes are similar between women with and without COVID-19, others reported that severity in pregnancy varies from asymptomatic to life-threatening [5]. Therefore, we aimed to report the maternal and neonatal outcomes of four pregnant women with COVID-19 in Tunisia. We also performed a literature review to identify COVID-19 infection maternal and neonatal outcomes.

## Patient and observation

### Case 1

**Patient information:** a 29-year-old woman returned from Algeria on July 3, 2020 (Table 1). She has no personal medical history and was 32 weeks pregnant G1P0A0. She was placed in a quarantine center on July 10 after returning from abroad.

**Table 1:** epidemiological and clinical characteristics of four Tunisian pregnant cases

Variables	Case 1	Case 2	Case 3	Case 4
Date of first positive RT-PCR	7^th^ July, 2020	13^th^ July, 2020	17^th^ July, 2020	29^th^ July, 2020
Age (years)	29	27	35	33
Gestational age on admission	32 weeks pregnant	20 weeks pregnant	24 weeks pregnant	16 weeks pregnant
Imported cases	Yes	Yes	Yes	Yes
Family cluster	No	No	No	No
Complications	No	No	No	No
Delivery mode	Vaginal delivery	Not yet	Cesarean delivery	Not yet

RT-PCR: reverse transcription-polymerase chain reaction

**Diagnostic testing:** a subsequent nasopharyngeal swab (based on reverse transcription-polymerase chain reaction (RT-PCR) testing) was performed, it returned positive on July 7 and thus was transferred to COVID-19 Monastir Center, a national containment center, dedicated for confirmed cases.

**Clinical findings:** the patient did not develop any symptoms or complications during containment and the pregnancy was very smooth.

**Follow-up and outcome:** after two consecutive nasopharyngeal swabs tested negative for severe acute respiratory syndrome coronavirus 2 (SARS-CoV-2) (on July 24 and 25), the case was home discharged, 2 weeks after the first positive RT-PCR. The patient gave birth on August 6, 2020, via a spontaneous vaginal delivery, the neonate presented no symptoms and no adverse outcomes.

**Patient perspective:** the patient adhered perfectly to the follow-up during the containment and throughout the pregnancy. The patient was happy to have an uncomplicated pregnancy and a healthy newborn.

**Declaration of patient consent:** the authors certify that they have obtained all appropriate patient consent.

### Case 2

**Patient information:** a 27-year-old Tunisian woman, returned from Ivory Coast on July 9, 2020 (Table 1). She was healthy, with no medical or gynecological history. She was 20 weeks pregnant G1P0A0 when she tested positive for COVID-19 on July 13, 2020.

**Clinical findings:** during the isolation period, the patient was asymptomatic and had a good course of pregnancy without any complications.

**Follow-up and outcome:** it turned negative on July 27 and 28 and the case was home discharged 15 days after the first RT-PCR and after two consecutive nasopharyngeal swabs tested negative for SARS-CoV-2 as recommended by the World Health Organization (WHO). The patient was followed up after being home discharged. The last contact was on October 26. She had a smooth pregnancy according to regular checkups and her latest ultrasounds.

**Patient perspective:** globally, the patient showed satisfaction with the healthcare in the containment center. Currently, her pregnancy is well monitored.

**Declaration of patient consent:** the authors certify that they have obtained all appropriate patient consent.

### Case 3

**Patient information:** a 35-year-old Tunisian woman, returned from Algeria on July 20, 2020 (Table 1). She was healthy, with no medical or gynecological history, and 24-week pregnant G2P1A0. When she arrived in Tunisia, she was confined in the national containment center. On July 27 (day 7 of containment), she tested positive for SARS-CoV-2. Thus, she was placed in the COVID-19 Monastir Center with frequent check-ups (temperature, capillary blood glucose, and arterial tension).

**Clinical findings:** she did not present any symptoms nor any complications.

**Follow-up and outcome:** the first control SARS-CoV-2 RT-PCR on August 3 was a false negative. It was revealed by a positive test outcome after 48 hours. It turned negative only on August 11 and 12 and the case was home discharged 16 days after the first RT-PCR and after 2 consecutive nasopharyngeal swabs tested negative for SARS-CoV-2. According to our phone calls follow-ups, she was in very good health as well as her pregnancy. Her cesarean delivery was scheduled on October 30 because of a previous cesarean section in labor. She delivered a healthy eutrophic boy.

**Patient perspective:** the patient was happy to have a pregnancy without complications and a healthy newborn.

**Declaration of patient consent:** the authors certify that they have obtained all appropriate patient consent.

### Case 4

**Patient information:** a 33-year-old Tunisian woman who was healthy and nonsmoker (Table 1). She was 16 weeks pregnant G1P0A0, working in an airline company. The company organized a COVID-19 screening campaign, and although being asymptomatic, she tested positive for SARS-CoV-2 on July 29, 2020. Therefore, she was put in the COVID-19 Monastir Center.

**Clinical findings:** the patient had no symptoms of infection.

**Follow-up and outcome:** this case did not receive any treatment during containment. The results of two continuous COVID-19 virus tests were negative for nasopharyngeal swabs. Thus, the patient was declared recovered on August 14, 2020 (16 days after the first RT-PCR). The patient left the containment center. She was contacted on October 26, she was 27 weeks pregnant. The case had a smooth pregnancy without any complications.

**Patient perspective:** the patient was satisfied with regular checkups in the national containment center and happy to have an uncomplicated pregnancy.

**Declaration of patient consent:** the authors certify that they have obtained all appropriate patient consent.

## Discussion

We reported data from four pregnant women with laboratory-confirmed COVID-19 pneumonia. Mothers´age were between 27 to 35 years and the gestational age when infection ranged from 16 to 32 weeks. The clinical characteristics of these four pregnant patients were similar to those of non-pregnant adults with COVID-19 infection. None of the four patients developed severe COVID-19 symptoms.

According to the latest studies, pregnant women can be completely asymptomatic as they can present severe forms indicating even hospitalization [4,5]. Indeed, Chen *et al*. study found that clinical, laboratory and radiological data were comparable to those observed in the general population [6]. However, some medical experts suggested that COVID-19 symptoms might be more serious among pregnant women compared to non-pregnant [7]. Pregnant women are more prone to infectious diseases because of many system changes [8]. Pregnancy gestational, immunologic, and physiological changes could increase infection susceptibility, and encourage rapid progression to respiratory failure. During pregnancy, the T-helper 2 (Th2) system dominates to protect the fetus and thus, predisposes mothers to viral infections, that are more contained by the Th1 system [9].

In the past two decades, severe acute respiratory syndrome coronavirus (SARS-CoV) and the Middle East respiratory syndrome coronavirus (MERS-CoV) epidemics were responsible for almost one-third of deaths between infected pregnant women [5]. In the 2009 H1N1 pandemic, pregnant women were found to be at a higher risk of complications and to be admitted to hospital than the general population (relative risk 4.3 (95% CI 2•3-7•8)) [10]. It makes sense so, to predict that SARS-CoV-2-infected pregnant women could be at a greater risk of severity and mortality compared to non-pregnant women.

A total of 24 studies, including 324 infected pregnant women with COVID-19, revealed that the most frequent COVID-19 onset symptoms were fever and cough. However, less common symptoms were myalgia, malaise, sore throat, diarrhea, and dyspnea. Lymphopenia and higher concentrations of alanine transaminase (ALT) or aspartate transaminase (AST) could also be detected [9].

Concerning neonatal and maternal outcomes, no complications occurred. Although, a systematic review demonstrated that the incidence of preterm births, low birth weight, C-section, and neonatal intensive care unit (NICU) admission was higher than the general population [11]. Case 1 had a vaginal delivery and case 3 a scheduled cesarean delivery due to a uterine scar. Decisions on delivery methods should be individualized. Vertical transmission of coronavirus from the pregnant woman to the fetus has not been established [8]. The International Society of Ultrasound in Obstetrics and Gynecology (ISUOG) guidance allows vaginal delivery if the labor onset is spontaneous and its progress is satisfying, the second stage should be shortened to avoid active pushing. Emergency cesarean delivery is indicated in case of acute organ failure, septic shock, or fetal distress [12]. To investigate COVID-19 intrauterine transmission, a recent study tested the amniotic fluid, umbilical cord, and newborn’s throat swab. SARS-CoV-2 was negative among all samples suggesting that no intrauterine fetal infection occurred [6,13]. In addition, COVID-19 was not detected in mother´s milk; so puerperal patients infected with SARS-CoV-2 are recommended to maintain breastfeeding [14].

## Conclusion

The current report does not present significant differences in the progression of the disease in the pregnant women´s group compared with the general women´s population. Following the new recommendations and update knowledge is crucial to ensure the highest quality of health care services among pregnant women. High quality studies are needed to establish the impact of COVID-19 on pregnant women.
